# How do search systems impact systematic searching? A qualitative study

**DOI:** 10.5195/jmla.2023.1647

**Published:** 2023-10-02

**Authors:** Andy Hickner

**Affiliations:** 1 alh4014@med.cornell.edu, Education and Outreach Librarian, Weill Cornell Medicine, New York, NY.

**Keywords:** Systematic reviews, user experience, thematic analysis, qualitative research, usability, expert searching, librarians, information retrieval, bibliographic databases, search engine

## Abstract

**Objective:**

Systematic reviews and other evidence synthesis projects require systematic search methods. Search systems require several essential attributes to support systematic searching; however, many systems used in evidence synthesis fail to meet one or more of these requirements. I undertook a qualitative study to examine the effects of these limitations on systematic searching and how searchers select information sources for evidence synthesis projects.

**Methods:**

Qualitative data were collected from interviews with twelve systematic searchers. Data were analyzed using reflexive thematic analysis.

**Results:**

I used thematic analysis to identify two key themes relating to search systems: systems shape search processes, and systematic searching occurs within the information market. Many systems required for systematic reviews, in particular sources of unpublished studies, are not designed for systematic searching. Participants described various workarounds for the limitations they encounter in these systems. Economic factors influence searchers' selection of sources to search, as well as the degree to which vendors prioritize these users.

**Conclusion:**

Interviews with systematic searchers suggest priorities for improving search systems, and barriers to improvement that must be overcome. Vendors must understand the unique requirements of systematic searching and recognize systematic searchers as a distinct group of users. Better interfaces and improved functionality will result in more efficient evidence synthesis.

## INTRODUCTION

Systematic searching is distinguished from other types of searching by its goals of exhaustiveness, transparency in reporting, and reproducibility [1]. It has increased in importance as a requirement of systematic review (SR) methodology and has led to increasing involvement in SRs by information specialists.

Systematic searches, typically conducted as part of systematic reviews, are notoriously time-consuming. One survey of systematic searchers found the average total time per project spent searching was 24 hours [2]. When non-search tasks are included, estimates of average time burden range from 23 [3] to 26.9 hours [4]. These significant costs in time and effort have prompted innovations including development of other review methodologies; novel search development methods [5]; and text mining [6].

Prior studies have evaluated whether search systems possess the required functionality. (For purposes of this article, I define “search systems” broadly to include bibliographic databases such as MEDLINE; platforms for searching databases, such as OvidSP; and other sources of data, such as trial registries.) Bethel and Rogers (2014) designed a checklist consisting of 55 “desirable or essential attributes” for what they described as complex searching, and used it to test 3 platforms (EBSCOhost, OvidSP, and ProQuest) [7]. They found that none “performed well for all of the attributes on the checklist.” More recently, Gusenbauer and Haddaway developed an alternative method to test a system's ability to support systematic searches [8]. They identified 28 search systems regularly used for evidence synthesis and tested each against a set of 27 criteria (14 of which were deemed essential), including controlled vocabulary, bulk export, and reproducibility. They found only 14 systems met the performance requirements for systematic searching.

In their review of current best practice in search strategy formulation, MacFarlane and colleagues identified shortcomings in the Boolean approach that currently predominates in systematic search interfaces, and proposed design principles to overcome them [9]. Other recent studies have examined the usability of automation tools in systematic searching [10,11]; reported the experiences of problems faced by novice systematic reviewers, and what strategies they used to overcome them [12]; quantified the amount of time invested in grey literature searching [2]; and documented challenges to grey literature searching [13].

### Research Rationale

As Gusenbauer and Haddaway have written, “To improve our finding capabilities, we urgently need to improve how we search and the systems we use” [1]. Prior research has generally focused on objective criteria, e.g., evaluation based on functional requirements, using a quantitative paradigm. Qualitative data collection methods such as interviews are commonly used in user experience research for learning about user needs and attitudes and exploring how they think about problems [14]; however, in-house user experience research is rarely published by the companies that conduct it. I undertook the present study to examine the effect of the well-documented imperfections of current search systems on the work of systematic searchers, using a qualitative approach. I sought to build upon prior studies by obtaining a fuller, richer, and more nuanced account of the process and context of systematic searching, and the role of search systems in both.

## METHODS

### Methodological Approach

I collected data through semi-structured interviews with systematic searchers, and analyzed it using the reflexive approach to thematic analysis (typically abbreviated as “reflexive TA”) developed by qualitative psychologists Virginia Braun and Victoria Clarke [15-17]. While Braun and Clarke developed their approach for use in psychology, its flexibility makes it useful for qualitative studies across the social sciences. In reflexive TA, the researcher aims to identify and describe patterns across qualitative data, represented by central organizing concepts (themes).

My approach to this project was critical-realist, assuming that people correct and supplant their perceptions through their own beliefs and other cognitive schema [18]. My epistemological position was contextualist, meaning it allows for the possibility of multiple accounts of reality and assumes that the researcher's own perspective influences their interpretation of data [17]. In user experience research, participants may provide subjective accounts that at times conflict with one another; to the extent that these accounts are consistent, it is insofar as they reflect a shared context (in this study, the context of systematic searching). My orientation to TA in this project was experiential and inductive. In experiential TA, participants' words are assumed to reflect their meaning and how they perceive reality; the researcher takes them at face value, so to speak, without extensively dissecting and analyzing for subtext what is being said [16]. Unlike approaches to thematic analysis that center “coding reliability” and which reflect a different philosophy of research, reflexive TA is premised on the idea that the subjectivity the researcher brings to data analysis is both inevitable and essential; therefore, reflexive TA does not incorporate elements such as multiple coders or calculation of intercoder agreement.

In a 2021 commentary, Gusenbauer and Haddaway provided a definition of systematic searching and proposed a model they described as the “Search Triangle,” which provided the theoretical framework for this study [1]. According to the Search Triangle, “efficient and effective search” only works when three elements are aligned: user goals; search heuristics (i.e., approaches to search); and search systems. This model informed the research design in terms of the focus of interview questions, which probed users' perception of the three elements vis-à-vis the search systems they used. The idea of mismatch between user goals and search system informed my analysis.

### Participants and Recruitment

Participants in this study were information specialists with experience working on at least one systematic review in the health sciences. I recruited participants via email using the Expert Searching listserv [19] and the listserv of the Medical Library Association (MLA) Systematic Review Caucus. In addition, an instructor from the University of Michigan's Systematic Reviews Workshop [20] forwarded the recruitment email to participants in recent Workshop cohorts. I used purposive sampling, in which participants are chosen based on pre-specified criteria that is relevant to the research objective [16]. Reflexive TA is not prescriptive about participant group size but suggests 6-10 “rich and detailed” interviews for a small project or 10-20 for a medium-sized project [21].

I asked potential volunteers to complete a brief eligibility screening using a Qualtrics screening form (see Appendix A). I then selected the sample of 12 volunteers from the 36 total who completed the screening form to ensure a range of experience levels, institutional affiliations, and geographic locations, and experience with systematic reviews in a variety of health sciences subject areas (e.g., medicine, public health, dental, etc.). Due to my history of professional service in health sciences librarianship, I was previously acquainted with some participants and familiar with the work of others; however, neither personal acquaintance nor familiarity with a participant were exclusion criteria. I contacted selected participants by email to consent and schedule the interview. No participants declined or were lost to follow-up.

### Data Collection

Data were collected via semi-structured Zoom interviews lasting 30-60 minutes. The first set of questions elicited information about the subject's experience with systematic reviews. I proceeded to a series of questions that captured the search systems that the subject uses regularly and that are most relevant to their work; questions about “pain points” in the search process; and questions about access to search systems. Some questions did not directly pertain to search systems, but rather were written to elicit potentially relevant information about the context and process of searching. The interview guide is available in Appendix B. The study was reviewed and approved by the Weill Cornell Medicine Institutional Review Board, #21-09023922.

Interviews were transcribed using Zoom's automatic transcription feature. Afterward, I uploaded the interview recordings to Otter.ai in order to add speaker identification. I conducted data cleaning to correct errors in the automatic transcription. I redacted potentially identifying data from the transcript, such as names, references to institutional affiliation, and the names of specific Cochrane work groups.

### Data Analysis

Data analysis followed the method outlined by Braun & Clarke for reflexive TA. I began by familiarizing myself with the data, reading each data item (individual interview transcript) and noting details of particular interest. I proceeded to code each data item using a flexible approach. In general, the concrete and specific nature of the matters discussed lent themselves to semantic (descriptive, surface-level) codes, supplemented with selective latent (implicit or conceptual) codes. After the entire dataset was coded, I reviewed the codes and used them to generate, define, and name initial themes, before reviewing and revising them into a final set of themes.

My analysis was informed by my position as a systematic searcher and librarian. I believe my “insider” perspective influenced my analysis and interpretation of the data in multiple ways. For example, it allowed me to explore technical details more deeply than a non-searcher might. It also influenced how I viewed the relationships between participants, their employers or customers, and vendors. Another aspect of my position was my prior experience with applied user experience (UX) research in both the classroom and the workplace. In addition to influencing my epistemology (as discussed earlier), it helped me navigate participants' subjectivity and their sometimes-conflicting accounts.

## RESULTS

Participant characteristics are summarized in Table 1.

**Table 1:** Participant characteristics

Country	USA (6 participants), Canada (3), UK (1), Japan (1), the Netherlands (1)
Type of organization	Academic library (8 participants); federal military (1); nongovernmental organization (1); academic medical center (1); independent consultancy (1)
Number of MEDLINE-indexed evidence synthesis publications*	Median 10; average 19.5; range 0-53
Years of SR experience (self-reported)	Median 9; average: 10.5; range 1-29

* Calculated through author search in PubMed on September 26, 2022, limited to articles indexed with the systematic review or meta-analysis publication type; excludes projects that did not result in publication in a MEDLINE-indexed journal.

I identified two themes: (Theme 1) Systems shape search processes, and (Theme 2) Searching occurs within the information market. Throughout this section, I use “searchers” as shorthand for “systematic searchers.” Certain quotes have been lightly edited for readability and conciseness (removing crutch words, unnecessary repetition, etc.). Quotes from the data are accompanied by the participant number in parentheses.

A significant amount of data from interviews was not directly relevant to my research rationale. When I asked about pain points, the first examples that came to mind for many participants were not directly related to search interfaces, but rather other aspects of the project, such as collaboration. These data nevertheless provide valuable context for understanding systematic searching and will be reported in a subsequent paper.

### Theme 1: Systems Shape Search Processes

Searchers navigate a variety of search systems to explore and retrieve bibliographic data. I use the related term “interface” to describe the platform-specific graphical user interface the user navigates in order to search a database. Aspects of search systems help or hinder their work. Process describes the tactics searchers devise and apply to mitigate an interface's limitations or leverage their affordances. Systems are the product of the information market (see Theme 2), which explains many of the pain points participants recounted.

Search processes are often idiosyncratic. Subjectivity was most evident in the resources selected for a project, e.g., choice of MEDLINE platform or which database to start in. Some participants who preferred PubMed to Ovid MEDLINE cited collaborators' perceived needs and preferences, such as easier sharing of exploratory search results. Which resources participants considered “core” depended on a range of factors, such as coverage, what they have access to, which they prefer to avoid, and the areas of specialization of their frequent collaborators.

Another area where subjective factors influenced the process was in search construction, i.e., single-line versus multi-line searches. Searchers were divided between the two camps, with sometimes strong opinions on either side about the merits of their preferred approach and the disadvantages of the other. Both approaches utilize Boolean logic (combining concepts using AND, OR, NOT), which present-day search interfaces almost universally use to structure queries. Only one participant in this study (07) discussed alternatives to the Boolean approach, echoing concerns that have been discussed elsewhere [9]:

**What I ideally want in an interface is the ability to combine concepts, and then play around with the concepts rather than the search terms. With most interfaces, that editing process where you recombine sets can be quite cumbersome to do and you can lose track of where you've got to… None of the interfaces are really good at letting you move large blocks of search terms around easily and recombine and see what is the impact of taking one concept out and adding another… Most of the main interfaces we use are limited when you're doing complicated searches and lots of set combination. And they don't show you visually how the combinations are working, which I think for a lot of people would be really helpful (07).**

This participant was the only one to discuss newer visualization-based approaches such as 2Dsearch [22] and PubVenn [23], which she saw as promising.

According to participants, factors that increased their efficiency included repeatedly working within the same subject area and/or with the same collaborators, and a higher volume of SR work. On the other hand, one participant noted that a heavy workload could adversely affect their capacity or willingness to try a new resource:

**I wonder how that impacts the number of databases (you search), how comfortable you feel playing around in a new one. Are you going to suggest one that you don't know when you start thinking about how much time it's going to take you to figure out those things?**

(10)

How regularly a platform is searched affects cognitive load: “Basically when I haven't used them in a while and I forget their little ways, is when it is a problem. The key to success is to use them all the time, and then you get the hang of it” (02); “I think some of the more time-consuming things are the databases that you don't use a lot, or ‘OK, how do I adjacency search in this database?’ and reteaching yourself” (11).

Although participants stated that they tried not to make choices about where to search based on the usability of a platform, several indicated it was a factor when a source seemed optional or was available on multiple platforms. These data provide evidence for the phenomenon of “resource selection bias,” which results from the failure to retrieve potentially relevant data when infrequently-used or user-unfriendly databases are avoided [24].

Interfaces mediate the searcher's interaction with each database. Many are not designed for systematic searching:

**Often these databases are designed to help clinicians quickly find a few resources. They're not designed to help somebody like me download thousands of records. So you're kind of going against the way the user interface has been designed (02).**

Participants echoed many previously reported concerns with grey literature platforms and other sources of unpublished studies [2, 13, 25], including those maintained by government agencies (e.g., the Defense Technical Information Center and the NASA Technical Reports Server), and registers of trials or economic evaluations: “Everyone hates grey literature” (12). Complaints included lack of required functionality; data that was not current as advertised; and challenges documenting grey literature searches, which reduces the level of detail in reporting in cases where the searcher's time is limited by the client's budget constraints. They described exporting results as a particular headache, requiring sometimes elaborate workarounds.

Participants emphasized the value of precision and control in interfaces. Databases with controlled vocabulary afforded greater precision; as one participant put it, “Anytime that I can't use PubMed for my starting point, it's gonna be more like buckshot” (03). The hierarchical nature of Medical Subject Headings (MeSH) helped teams refine the scope of questions. While some described leveraging better subject-specific controlled vocabulary in specialized databases, others noted more granular controlled vocabulary and indexing sometimes posed its own challenges:

**(S)ometimes it's just annoying how much Embase brings back, the level of granularity of the indexing… (T)ranslating that to other databases, once you have this list of 300 synonyms? It makes me wonder about, you know, diminishing rates of return (08).**

One participant described projects that were abandoned, then revived years later:

**There are now new subject headings… You're not even with the same search, you're not getting the same results… Two of them, when we went through what was going to change, said, no, we're going to start from square one. And we're going to do the whole rescreening right from the beginning, because there's so much change that it would never be replicable (05).**

Another worried that the shift to automated indexing in MEDLINE and Embase comes at a cost to precision: “If it's been automatically added, then the computer thinks for you, and then sometimes they use an index term that's really not relevant for the article” (06).

In some systems, participants felt the available functionality is useful but has poor usability (particularly in the case of the Wiley interface for the Cochrane Library); in others, they reported struggling when the appropriate configurations for systematic searching conflicted with those for other types of users. They complained that core functionality in some systems was unreliable or felt unpredictable. Multiple participants cited the tedious process of translation between databases as a pain point. While an automation tool for translation (Polyglot) exists [26], some participants indicated they translate by hand, in part because Polyglot still requires significant human quality control and cannot yet translate controlled vocabulary across databases.

One subtheme could be described as “A culture of sharing.” Several participants described drawing on the global community of systematic searchers for help navigating pain points. Participants cited examples such as sharing Word macros for translation, Twitter discussions, sharing workarounds for exporting from preprint servers, and sharing filters and methodological tutorials on the Internet:

**Hanging filters on the Web is another big workaround… either things that have way too many words to think up, or they're things that are really hard to get to… One of the reviews that I did for occupational therapy, because it was OT and musicians… Once you have figured out what the list of musicians and musical instruments is that might occur in Ovid MEDLINE, there is absolutely no point in anybody else ever figuring that out again (05).**

Participants' accounts of their workarounds for pain points revealed unmet needs and opportunities for innovation. They identified the need for potentially useful tools that do not yet exist, including automatic conversion of single-line searches to multi-line ones; conversion between controlled vocabularies; easy import and combination of filters from external sources; and effective and reproducible federated search across multiple databases: “I'll tell people that sometimes when I'm going over the process, one of these days, there's going to be one search engine that's going to search all of these databases, but we are not there yet” (12).

### Theme 2: Systematic Searching Occurs within the Information Market

**We're always at the beck and call of our budget as a librarian. I think that's just going to be the way it is until the end of time, unfortunately… budget constraints are omnipresent (11).**

Theme 2 echoes one of the frames in the ACRL Information Literacy Framework, “Information has value”: “Information possesses several dimensions of value, including as a commodity… (S)ocioeconomic interests influence information production and dissemination” [27]. Evidence synthesis is costly both in labor and financially since it usually relies upon subscriptions to proprietary systems and journals, and it requires compromises based on scarcity and constraints. One participant described how academic health sciences librarians compete for budgetary resources with other disciplines and categories of users: “(The way) the decision-making power is allocated at [my employer's] library… it's over-representation of humanities and social sciences because those are the larger staff libraries” (10). No participant had the luxury of access to every platform/database combination they preferred.

At times, searchers are successful in advocating for keeping or adding a resource due to its utility for evidence synthesis:

**Ovid is one of the most expensive platforms that you can license. A couple years back, there was some question as to what we were going to do there. And we rallied, that we needed it, there was just no way we could do our work without it. And it was kept (11).**

The “cost savings” from inferior platforms may not be worth it:

**We're trying to make a point of dictating that when decisions like those come up, they need to consult with the librarians more heavily. Because some of those decisions, even if they look better on paper… there are other factors to consider, like interoperability, ease of use, who can access it… (04)**

Scarcity of budget and time impacted database selection. As the independent consultant put it, “A lot of it is driven by what my clients subscribe to, and what their budget is” (07), while an academic librarian remarked, “I'm much more interested on potential return on investment now, investment being my search time” (01).

Vendor-searcher relations shape and are shaped by the information market. Vendors may hold monopolies over key databases, with implications I will explore later. A database may be necessary for a given topic even if it has significant limitations for systematic searching: “I think it's just grit your teeth and get through it, if it's a relevant source” (03). Participants described conventions and best practices that nevertheless were not implemented uniformly across platforms for basic aspects such as syntax, controlled vocabulary lookup, field definitions, export format, and search history query order, as well as lack of alignment in functionality such as metadata export from PubMed to SR software. This lack of standardization wastes searchers' time and effort.

Participants' perceptions of vendors often reflected brand experience, an aspect of user experience which Law and colleagues (2009) defined as “not only interaction with the branded products, but interaction with the company, its products and services… Brand experience affects the user experience when you interact with the product: you forgive flaws for a loved brand and blame loudly the flaws in the products of a bad brand” [28]. Participants appreciated responsiveness to customer requests, such as adding the ability to edit lines or insert them in a non-sequential manner in OvidSP; proactive communication of changes; and learning from customer representatives about functionality they were unaware of. On the other hand, they described struggling to get platform owners to respond to their needs:

**EBSCO in particular is just a horrifying mess… We actually have a running list of what's wrong with EBSCO… (which) we periodically send that to the reps who heave it in the garbage or something… (W)hen the Canadian Health Libraries Association Conference was in Vancouver, I stood at the EBSCO desk and said, I want this this and this fixed. And the guy said, Oh, you librarians and your systematic reviews… (More recently) I sent the rep the entire list. And she said, Oh, well, I'll take this forward. Nothing, absolute radio silence. They are just so totally unresponsive (05).**

Several participants cited EBSCOhost as a particularly vexing platform, sometimes complaining at length. (Several also expressed frustration with the Wiley interface for the Cochrane Library.) In their 2014 evaluation of OvidSP, ProQuest, and EBSCOhost, Bethel & Rogers found that “only one (OvidSP) performed well on the majority of functions required for complex searching,” noting, “As the other two host providers have sole commercial rights to key health databases, it is imperative that the platform is capable of running complex searches for systematic reviews” [7]. Nine years later, these interviews indicated a general consensus that EBSCOhost had made little progress toward that imperative, and a sense of lack of control over the search which results in a lack of trust in the system (to use MacFarlane and colleagues' definitions of these concepts) [9]. When a platform owner holds a monopoly over an essential database (in this case, CINAHL), it may explain the unresponsiveness to customers that some participants reported.

Participants also expressed concern that PubMed had begun to deprioritize systematic searching:

**This message has come across very loud and clear from NLM, which is that their audience is the average searcher, and they are not interested in advanced searchers, and unfortunately if that is the way that things continue, more of us may get pushed over into Ovid MEDLINE if we can afford it (01).**

**It's sort of simplified down in the last iteration of development. And it lost a lot of the features that were useful… You can no longer export records in XML, unless you use the API. It's just – a lot of the features that it needs to become a systematic review interface that just aren't there really (07).**

Recent changes to the PubMed search algorithm, including automatic term mapping, have decreased the reproducibility of older searches [29]:

**Even if I take a search that I did a few years ago in PubMed and try to do it now, it acts differently… All these interfaces are moving away from that more, and more relying on algorithms and other behind the scenes things. I don't care for that, and I don't think that it is a good shift. But I think the horse has left the barn on that one (04).**

Finally, participants noted the constant change in the evidence synthesis environment, the pace of which was accelerated by the COVID-19 pandemic. One participant described how and why their organization created its own COVID-19 literature database early in the pandemic:

**For review teams, I needed information straightaway. And, you know, building searches across platforms was time-consuming, time that we didn't have… (E)arly on, we realized we have to put in the preprints. Because every day somebody would come up with “Have you seen this preprint?” (09)**

In short, Theme 2 illustrates the mismatch between users' goals and systems that persists in systematic search. Is it possible that vendors do not perceive a market incentive for prioritizing systematic search? It also illustrates how even where a system may offer the best functionality, searchers may not have subscription access to it. Theme 2 encompasses a variety of challenges searchers face, including scarcity (both financial and of time), market forces such as lack of competition among vendors, and differences between the needs of systematic and non-systematic searchers.

## DISCUSSION

The main findings of this study are the two themes: Systems shape search processes (Theme 1), and systematic searching occurs within the information market (Theme 2). In terms of Theme 1, this study complements and extends prior evaluations of search functionality across search systems that utilized quantitative approaches [7,8] by providing empirical data from searchers illustrating how current system limitations impact searching in practice. Participants' subjective accounts and descriptions of their practice provide deeper and more complex insight into the user experience of searching. Consistent with the “Search Triangle” model [1], participants reported inefficiencies that in part result from the mismatch between user goals and systems. Translation is one example of a pain point described by participants and also previously cataloged by MacFarlane and colleagues [9]. Likewise, some of the solutions they imagined - for example making it easier to move around and recombine concept blocks - are consistent with design principles MacFarlane and colleagues proposed. For example, principle 1, “Support transparency in mapping,” would facilitate rearrangement and re-combination of concept blocks, making the high-level structure of the search easier to decipher while reducing mistakes in line number combination. Searchers and platform owners might collaborate to explore ways to implement these principles across systems.

This study complements and extends prior work on the topic that used survey [2] and case report [13] methodologies. These prior studies described the challenges of grey literature retrieval, improvement to which is perhaps the most urgent priority revealed in the interviews. While the Cochrane Central Register of Controlled Trials (CENTRAL) provides access to trials data with better search and export functionality, it is not comprehensive enough to be the only means of identifying unpublished trials [30]. There is a clear demand for grey literature search systems that better meet the needs of systematic searchers.

Theme 2 is relatively novel insofar as it focuses on aspects that have not been examined in detail in prior research and commentary on systematic searching; namely, how searchers are constrained by access to specific search systems, and factors that determine access, such as budget and competition with other types of users. I have argued that limitations persist on some platforms in part because a vendor has a monopoly on the database (e.g., CINAHL) or has chosen to prioritize the needs of other types of users.

### Limitations, Strengths, and Future Directions

One limitation of this project is the relative homogeneity of the participant group: mostly North American and native speakers of English, reflecting the venues through which I recruited. A second limitation is that I did not have the benefit of a supervisor experienced in reflexive TA, who might have helped me interrogate my coding choices and assumptions more thoroughly and reflectively. I attempted to mitigate this by availing myself of the extensive publications providing guidance on the reflective TA methodology [16,17]. Strengths include a group of participants that represents multiple types of institutional settings, ranging from academic to nongovernmental to independent consultant, and that have experience in systematic reviews across a wide range of health sciences domains.

Future research could attempt to quantify phenomena discussed by this study's participants: for example, measuring the amount of time it takes to translate a search, or comparing the efficiency of searching a specific database on different platforms. Usability testing to evaluate different approaches to functionality across platforms could be conducted with a different group of participants, such as novice systematic searchers or non-information specialists, which could generate very different data.

In terms of implications for practice, librarians involved in collection development may find it useful to review participant observations regarding vendors' responsiveness to customer requests (or lack thereof). An unresolved question is how to get vendors to address the myriad problems with their interfaces and what the barriers to problem-solving are. This study provides evidence that searchers and their managers could cite as they advocate for their needs in collection development within their institutions. The summary of pain points and workarounds could also inform training curricula for novice systematic searchers.

Finally, innovation-minded platform owners should recognize systematic searchers' need for better, more standardized search systems. In this study, participants have provided vendors with a “wish list” of functionality they should seek to implement, such as easier combination and rearrangement of conceptual search blocks, larger export limits, and more standardized interfaces. Working with their customers, vendors can invest in improvements to usability and functionality of search systems; recognize the differences between systematic searchers and other searcher types; draw on this and prior studies on systematic searching to identify opportunities for product innovation; and continue to develop new tools to aid systematic searching.

## CONCLUSION

Thematic analysis of data from interviews with systematic searchers generated two overarching themes relating to search systems: systems shape search processes (Theme 1), and systematic searching occurs within the information market (Theme 2). In theme 1, participants detailed the limitations they encounter in search systems that were not designed for systematic searching, and how they navigate those limitations. They described workarounds which represent unmet needs and opportunities for improvement. Theme 2 illustrates the ways in which the “information market” constrains searching and how platform owners fail to prioritize systematic search functionality. Together these themes suggest priorities for future research involving search systems and systematic searching. This study provides evidence for systematic searchers and libraries to help them advocate for their needs, and for platform owners on why and how to better address those needs.

## Data Availability

Data associated with this article are available in the Qualitative Data Repository at https://doi.org/10.5064/F68AAMX3.

## SUPPLEMENTAL FILES

**Appendix A:** Participant recruitment screening form

**Appendix B:** Interview guide
