# Supplementary material for: How do search systems impact systematic searching? A qualitative study
Source: J Med Libr Assoc. 2023 Oct 2;111(4):774–82. doi: 10.5195/jmla.2023.1647 (PMC10621724; doi:10.5195/jmla.2023.1647)
Supplement: Supplementary file 1 — Appendix A: Participant recruitment screening form [file jmla-111-4-774-s01.pdf]

Appendix A: Participant recruitment screening form

[Link to pre-screening form (Qualtrics): https://bit.ly/3f8Hx4E](https://bit.ly/3f8Hx4E)

Q1 First name

____

Q2 Last name

____

Q3 Email address

____

Q4 Employer (or primary institutional affiliation). (If none, state "N/A")

____

____

Q5 Number of SR projects that you have completed and delivered search results for:

____

Q6 Number of SR projects that have resulted in a publication:

____

Q7 Have you collaborated on systematic reviews in any of the following disciplines? (Select all that apply)

- ☐ Clinical medicine (1)
- ☐ Dental (2)
- ☐ Global health (3)
- ☐ Health informatics (4)
- ☐ Nursing (5)
- ☐ Public health (6)
- ☐ Pharmaceutical (7)
- ☐ Psychiatry &/or mental health (8)
- ☐ Other allied health (e.g. occupational, physical, or speech therapy, etc.) (9)

Q8 In the event I am not selected to participate in this study, I am interested in participating in future research into user experience of search interfaces by the study team.

- ○ yes (1)
- ○ no (2)
