# Supplementary material for: How do search systems impact systematic searching? A qualitative study
Source: J Med Libr Assoc. 2023 Oct 2;111(4):774–82. doi: 10.5195/jmla.2023.1647 (PMC10621724; doi:10.5195/jmla.2023.1647)
Supplement: Supplementary file 2 — Appendix B: Interview guide [file jmla-111-4-774-s02.pdf]

Appendix B: Interview guide

The first set of questions in the interview will elicit background information about the subject's experience with systematic reviews. It is followed by a series of questions that will capture the specific databases and platforms that the subject uses regularly and that are most relevant to their work; questions about "pain points" in the search process that will shed light on usability concerns that are currently unaddressed in one or more search interfaces; and questions about the degree to which searchers' needs are reflected in their institution's collection development decisions.

(The following questions may be supplemented with follow-up questions and probes if warranted.)

Thanks for agreeing to meet with me today. Today's interview should take between 30 minutes to an hour. The goal of this study is to describe the needs of librarians working on systematic reviews in the health sciences and to evaluate the extent to which they are being met by online search interfaces. During the interview, I may ask you to use Zoom's screenshare feature to share examples of artifacts relevant to that question – for example, project documentation, search strategies and/or narratives, database interfaces, and software tools.

As was mentioned in the consent form you signed, you are free to withdraw your consent at any time; you can choose to discontinue the interview at any time; and you can opt not to answer specific questions if you choose. All the data from this interview will be deidentified before analysis.

Our interview will be recorded – do I have your permission to record?

1. Tell me about your experience working on systematic review (SR) projects.

a. How long have you been working on SR projects?

b. How many have you completed and delivered search results for?

c. How many have resulted in publication?

d. For what disciplines have you worked on systematic reviews? (e.g. clinical medicine, psychiatry, nursing, allied health, health informatics, etc.)

2. What platforms do you use to search MEDLINE for SR projects?

3. What other platforms/databases do you routinely search for SR projects?

4. What platform do you typically begin your search with?

5. What methods &/or tools do you use to translate the initial strategy to other databases?

6. What platforms do you find easiest and/or best-suited for systematic searching? What interface features do they have that are especially helpful?

Pain points

7. In your most recent SR project, what were the pain points in the process? (eg constructing queries, syntax, citation export)

8. Are there any databases/platforms that give you trouble? Why?

9. Are there any topics that you find to be more difficult to search because of the platforms required for that topic? (e.g. psychiatry, nursing, allied health, health informatics, etc.)

10. Describe any workarounds you use in response to these pain points.

11. Does the ease of searching a particular source factor into your selection of which sources to search?

Collection development

12. In your opinion, to what extent are your preferences regarding platforms considered when your library decides which ones to license? (i.e. when there is more than 1 platform available for a database)
13. Are there any databases or platforms your library does not license that you wish it did? What useful coverage and/or functionality would it provide that you currently lack?

Wrap-up

14. Is there anything I haven't asked about that you'd like to share with me?
